# Optimizing Scoring Function of Protein-Nucleic Acid Interactions with Both Affinity and Specificity

**DOI:** 10.1371/journal.pone.0074443

**Published:** 2013-09-30

**Authors:** Zhiqiang Yan, Jin Wang

**Affiliations:** 1 Department of Chemistry & Physics, State University of New York at Stony Brook, Stony Brook, New York, United States of America; 2 State Key Laboratory of Electroanalytical Chemistry, Changchun Institute of Applied Chemistry, Chinese Academy of Sciences, Changchun, Jilin, China; Wake Forest University, United States of America

## Abstract

Protein-nucleic acid (protein-DNA and protein-RNA) recognition is fundamental to the regulation of gene expression. Determination of the structures of the protein-nucleic acid recognition and insight into their interactions at molecular level are vital to understanding the regulation function. Recently, quantitative computational approach has been becoming an alternative of experimental technique for predicting the structures and interactions of biomolecular recognition. However, the progress of protein-nucleic acid structure prediction, especially protein-RNA, is far behind that of the protein-ligand and protein-protein structure predictions due to the lack of reliable and accurate scoring function for quantifying the protein-nucleic acid interactions. In this work, we developed an accurate scoring function (named as SPA-PN, SPecificity and Affinity of the Protein-Nucleic acid interactions) for protein-nucleic acid interactions by incorporating both the specificity and affinity into the optimization strategy. Specificity and affinity are two requirements of highly efficient and specific biomolecular recognition. Previous quantitative descriptions of the biomolecular interactions considered the affinity, but often ignored the specificity owing to the challenge of specificity quantification. We applied our concept of intrinsic specificity to connect the conventional specificity, which circumvents the challenge of specificity quantification. In addition to the affinity optimization, we incorporated the quantified intrinsic specificity into the optimization strategy of SPA-PN. The testing results and comparisons with other scoring functions validated that SPA-PN performs well on both the prediction of binding affinity and identification of native conformation. In terms of its performance, SPA-PN can be widely used to predict the protein-nucleic acid structures and quantify their interactions.

## Introduction

Precise regulation of the biological activities within cells is accomplished by biomolecular recognition which mainly involves three major biological macromolecules, i.e. protein, DNA and RNA. The protein-nucleic acid (protein-DNA and protein-RNA) recognition is essential to the regulation of gene expression at every level of the central dogma of molecular biology, including replication, transcription and translation of genetic information [1]. Determination of the structures of the specific protein-nucleic acid recognition and insight into their interactions at a molecular level are vital to understanding the regulation on a genomic scale [2]. The knowledge of which would be also enormously useful for a variety of biological and medical applications [3]–[8].

Although the structures of individual biomolecules are increasingly well determined and structural studies of the biomolecular complexes have been very active in the last decade, three-dimensional atomic structures of many biomolecular complexes are still difficult to determine due to the technical challenges of the experimental approaches [9]–[11]. As an alternative, computational approaches can complement existing experimental data and be applied to the structural prediction of biomolecular complexes [12]. The field of protein-protein docking has achieved substantial progress over the last decade as witnessed by the CAPRI (Critical Assessment of Predicted Interactions) [13, 14]. However, the progress for the protein-nucleic acid docking, especially the protein-RNA docking, lags behind due to the lack of reliable scoring function of protein-nucleic acid interactions. Previously, structural information was used extensively to derive scoring functions for successful predictions of protein structures, as well as protein-ligand and protein-protein interactions. Given the rapid growth in the number of solved protein-nucleic complex structures recently [15], it is natural and urgent to develop an accurate scoring function of protein-nucleic acid interactions, general for both protein-DNA and protein-RNA interactions.

For biomolecular functions, highly efficient and specific biomolecular recognitions are required to satisfy both the stability and specificity. The stability is determined by the affinity of the complex while the specificity is controlled by the partner binding to other competitive biomolecules discriminatively. The current scoring functions of biomolecular recognition [16], [17], whether force-field based, empirical, or knowledge-based scoring functions, mainly focused on improving the ability of predicting the known binding affinities observed in experiments as accurately as possible. The strategy of developing these scoring functions seeks to optimize the stability based on the combination of energetics and shape complementarity, but are often lack of the considerations of the specificity. In the cell compartment, biomolecules are required to function by interacting with a small number of partners rather than the myriad of others. The naturally occurred biomolecular recognition is just a very small part of all possible interacting complexes [18]. According to the Boltzman distribution (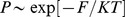), the equilibrium population is exponentially dependent on the binding free energy. A gap in binding free energy or affinity leads to significant population discrimination of the native complex against alternative ones [19]–[24], which is the requirement for the proper functions of the specific biomolecular recognitions in cell. Recent works taking the consideration of specificity into the computational design and optimization of interface interactions has achieved a few successful applications [6], [19]–[23], [25]–[29]. These works designed and optimized the interactions that seek to stabilize the desired structures and also destabilize the competitive structures. Thus, the accurate scoring function of biomolecular interactions should satisfy the criteria that the stability of the specific complex is maximized while the stability of alternative complexes is minimized, which can guarantee both the stability and the specificity for the functional biomolecular recognition.

**Figure 1:**
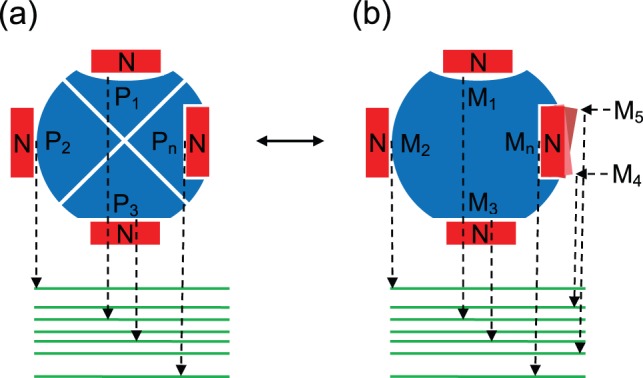
Schematic view of illustrating the equivalence of conventional specificity to intrinsic specificity. (A) The same nucleic acid (N, red) binding with multiple protein receptors (blue, 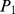 to 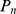), showing the conventional specificity as the gap in binding affinity of the nucleic acid binding to the specific protein receptor (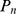) in discrimination against other protein receptors. The binding affinities are represented with corresponding energy spectrum (green). (B) The same nucleic acid (N, red) binding on a large protein receptor thought as the multiple different receptors linked together (blue) with multiple binding modes (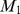 to 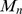), showing the intrinsic specificity as the gap in binding affinity of the native binding mode (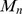) in discrimination against other binding modes.

The reason that the specificity usually was not taken into account previously in the development of the scoring functions is that the description of binding specificity was challenging to quantify. The conventional definition (Figure 1A) of specificity is the ability of a biomolecule to specifically bind to its own partner against other competitive partners, namely the relative difference in affinity of one specific biomolecular complex to others [24], [30]. The definition of conventional specificity is simple but the quantification of conventional specificity is challenging since it requires that the set of competitive complexes are not too large and the affinities are already known. This makes the practical quantification of the specificity impossible due to the incomplete information on the competitive alternatives.

To overcome the challenge, we have proposed a novel concept named as intrinsic specificity (Figure 1B) [19]–[23]. Here, we expand the concept to the protein-nucleic acid interactions. In particular, the intrinsic specificity of protein-nucleic acid binding refers to the preference in affinity of a nucleic acid binding to its protein receptor with a preferred pose over other poses (Figure 1B). Imagining the N- and C-terminus of multiple protein receptors are linked together, leading to an effective single large protein receptor. Under the assumption that the protein receptor is large, the conventional specificity of discrimination of a nucleic acid binding to its protein receptor against other proteins can be transformed to the intrinsic specificity that the nucleic acid binds to the large receptor with a preferred pose over other poses. By applying this concept, we have developed scoring functions for the interactions of bimolecular recognition, including protein-ligand interactions [21] and protein-protein interactions [22]. Also, the connection between the intrinsic specificity and conventional specificity was validated by studying a drug-target model [23], where the conventional specificity is correlated with the intrinsic specificity.

According to the theory of energy landscape [19]–[21], [31]–[39], the binding process of biomolecules can be visualized and quantified as a funnel-like energy landscape towards the native binding state with local roughness along the binding paths. The native pose of protein-nucleic acid complex is the conformation with the lowest binding energy and the energies of the conformation ensemble follow a statistical Gaussian-like distribution. The intrinsic specificity ratio (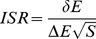, where 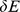 is the energy gap between the energy of native conformation and the average energy of conformation ensemble, 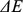 is the energy roughness or the width of the energy distribution of the conformation ensemble, and 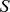 is the conformational entropy) can be defined to quantify the magnitude of intrinsic specificity. With computationally generated non-native poses (decoys), the ISR can be readily obtained. Therefore, without evaluating the conventional specificity through exploring the whole set of competitive partners, ISR physically provides a quantitative measure of the binding specificity.

In this work, based on our practical quantification of binding specificity, we have designed an optimization strategy to maximize both the affinity and specificity of native binding mode simultaneously for developing the scoring function of protein-nucleic acid interactions. The optimization strategy is to adjust the statistical knowledge-based potentials of atom pairs by iteration until the scoring function can effectively discriminate the native binding pose against the decoys. The flow of developing procedures is shown in Figure S1 in File S1. We have tested the derived scoring function of protein-nucleic acid interactions (SPA-PN) via the performance on the prediction of binding affinity and the identification of native binding pose. The performance of SPA-PN demonstrated that the quantified specificity is necessary to be incorporated into the optimization of scoring functions of protein-nucleic acid interactions.

## Materials and Methods

### Preparation of the datasets

#### Training dataset

The requirement of optimizing the knowledge-based statistical scoring function is to train a set of known structural data. The training dataset of protein-nucleic complexes for developing SPA-PN were extracted from the database NPIDB (Nucleic Acids-Protein Interaction DataBase) [15], [40]. NPIDB contains information derived from structures of protein-DNA and protein-RNA complexes deposited in the PDB (Protein Data Bank) [41]. To obtain a relatively high quality set of protein-nucleic complexes for the training dataset, X-ray structures with resolutions better than 3.0Å for the protein-DNA complexes and 3.5Å for protein-RNA complexes were selected. Entries with more than 3 DNA or RNA chains, or the number of heavy atoms of any chain less than 100 were discarded. By removing the entry overlaps with the testing datasets below, the resulting training dataset contains 1555 complexes, including 1221 protein-DNA structures and 334 protein-RNA structures (Table S1 in File S1).

#### Testing datasets

To validate the performance of a novel scoring function, two kinds of tests are needed. First, to evaluate the ability of SPA-PN on predicting the binding affinity. SPA-PN was tested on a dataset of protein-DNA complexes with known experimental binding affinities. This dataset for binding affinity prediction was employed from the binding database which is a modified version of Zhang *et al.* [42] and was used by Donald *et al.* [43] and Xu *et al* [44]. The dataset (named as testing dataset1) contains 30 protein-DNA complexes (Table S2 in File S1).

Second, in order to evaluate the ability of SPA-PN on discriminating the native conformation from decoy conformations, SPA-PN was tested on our collected benchmark of protein-nucleic acid complexes. The collected benchmark for binding pose prediction was obtained from available benchmarks of protein-nucleic acid complexes. It combines two protein-DNA benchmarks and two protein-RNA docking benchmarks. The first protein-DNA benchmark obtained from the PDIdb (Protein-DNA Interface database) [45] contains 246 representative protein-DNA interfaces out of 922 entries collected in the PDIdb. The second protein-DNA benchmark [46] contains 47 complexes which covers almost all major groups of DNA-binding proteins according to the classification of Luscombe *et al* [47]. The first protein-RNA benchmark [48] contains 45 complexes covering all major groups of protein-RNA complexes according to the classification of Bahadur *et al* [49]. The second protein-RNA benchmark [50] is an extended set of the first one and contains 106 protein-RNA complexes, it was obtained from both experimental and homology modeling data. This collected testing dataset were also filtered with the criteria as composed on the training dataset. In addition, one entry was kept if there are overlaps among the different benchmarks. The final collected dataset (named as testing dataset2) contains 315 complexes, including 232 protein-DNA structures and 83 protein-RNA structures (Table S3 in File S1).

#### Docking decoys

For the optimization of SPA-PN, an ensemble of decoys for each complex are needed to calculate the ISR for specificity and carry out the iteration algorithm. The RosettaDock v3.4 was taken as the structure optimization and docking tool [51], [52] to generate the decoys. Three steps were performed. First, each docking partner of the complex was prepared in isolation for optimizing their side-chain conformations prior to docking using the prepacking protocol. Second, the prepacked complexes were relaxed and minimized with high resolution by the refinement protocol. Third, the refined structures were taken as the starting structures for the docking using the local docking perturbation protocol. The smaller partner was defined as the docking ligand in the complex and the other was assigned as the receptor which was kept fixed during docking. 1000 orientations for each complex were generated by docking. Other docking parameters were set as default. The generated decoys are structured diversely to explore the underlying binding energy landscape.

### Derivation of knowledge-based statistical potentials

#### Observed statistical potentials

The knowledge-based scoring function consists of a set of statistical distance-dependent atom-pair potentials to quantify the interactions. Normally, the observed atom-pair potentials were directly derived from the Boltzmann relation widely applied in the derivation of knowledge-based statistical potentials for the protein-ligand, protein-protein and protein-nucleic acid interactions [53]–[57], the Boltzmann relation is written as
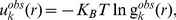
(1)where 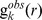 is the observed atom pair distribution function quantified by



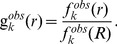
(2)

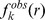
 is the observed number density of atom pair 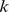 within a spherical shell between radius 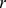 and 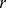
+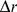. It can be directly extracted from the structural database of protein-nucleic acid complexes. 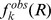 is the number density within the sphere of the reference state where there are no interactions between atoms. It was obtained based on the approximation that the atom-pair 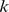 is uniformly distributed in the sphere of the reference state [58]. Respectively, they were calculated as
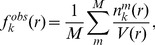
(3)

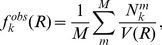
(4)where M is the total number ( = 1555) of training protein-nucleic acid complexes (Table S1 in File S1), 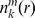 and 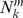 are the numbers of atom pair k within the spherical shell and the reference sphere for a given protein-nucleic acid complex m, where 
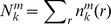
. 
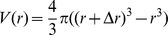
 and 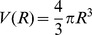 are the volumes of the spherical shell and the reference sphere, where 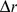 is the bin size and 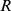 is the radius of sphere. 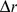 and 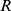 are set as 0.3Å and 8.2Å, respectively. In total, there are 20 spherical shells with bin size 0.3Å from the shortest radius 2.2Å. Based on the definition of atom type by SYBYL [59], 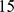 atom types were used to cover the heavy atoms involved in protein-nucleic acid interactions (Table S4 in File S1), these atom types were converted from PDB files by OpenBabel [60]. A cutoff ( = 600) of total occurrences for atom pair k (
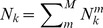
) was employed to neglect the contribution from the atom pairs with statistically insufficient occurrences. This lead to 95 effective types of atom pairs for the protein-nucleic acid interactions (Table S5 in File S1). There are 1900 types of interaction pair by multiplying the number of atom pairs ( = 95) and the number of shells ( = 20). In addition, if the atom pair has no occurrence in a particular spherical shell, the corresponding pair potential was set as the van der Waals interaction within this shell.

The observed statistical potentials from the known structures has its limitations as the statistical potentials extracted from equation (1) is not exactly the expected potentials that nature employs to stabilize the complexes [61]. The origin of this problem is attributed to the construction of the reference state where the atom pairs are uniformly distributed and independent of each other [58]. In reality, the protein-nucleic acid interactions involve the excluded volume, sequences and connectivity. Thus the observed statistical potentials are generally not equal to the expected potentials [62].

#### Expected statistical potentials

To circumvent the reference state issue and improve the statistical potentials, earlier efforts [42], [61], [63]–[65] have taken different approaches to optimize the statistical potentials. An effective way is to take into account both native and nonnative conformations (decoys) [61], [63], [65] based on the energy landscape theory that the native conformation should be sufficiently favored over alternative nonnative structures thermodynamically. However, these earlier works hasn't combined both the specificity and affinity to discriminate native conformation over nonnative conformations. In our recent papers on the study of the protein-ligand [21] and protein-protein interactions [22], we considered the importance of both the affinity for stabilizing the native conformation and the specificity of discrimination over nonnative conformations, and combined them into the optimization processes of scoring function. Here we expand this concept to optimize the statistical potentials of protein-nucleic acid interactions.

The expected statistical potentials are calculated similarly as the observed statistical potentials, which is
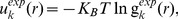
(5)where 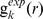 is the expected atom-pair distribution function from all the native and non-native conformations, which is



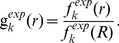
(6)Our aim is to obtain a scoring function that can significantly favor the native conformation over all other decoy conformations, so that the native conformation dominates the ensemble of conformations according to the Boltzman distribution. Considering the population discrimination of the native and nonnative conformations, the expected number density of atom-pair 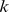 was calculated with Boltzmann-averaged weighting over the ensemble of conformations [22], [61], [65], that is
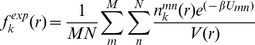
(7)

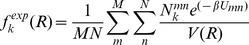
(8)where M is the number of native complexes mentioned above and N is the number of total conformations ( = 1001 including the native conformation and decoys) for each complex m. n represents the nth generated decoy of the complex m. 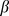 is a constant analogous to the inverse of temperature and set as 0.1. The resulting 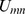 is the potential which is supposed to be able to discriminate the native conformation against decoys. As discussed, both the stability and specificity are the requirements to form an efficient and specific functional complex. Thus, 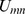 is designed to take into account of both affinity and specificity optimized through parameterizing the affinity (E) and specificity (ISR), which is given by



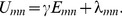
(9)

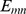
 is the energy score of the protein-nucleic acid conformation (nth decoy of the complex m) by summing over all the expected interatomic pair potentials among the interface, representing the affinity of the protein-nucleic acid conformation, 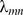 is the ISR representing the specificity of the protein-nucleic acid conformation [19]–[21]. 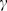 is a parameter which balances the values of 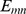 and 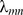 and set as 0.1. 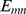 and 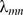 are calculated as
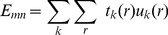
(10)

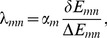
(11)where 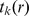 represents the occurrence times of the atom-pair interaction between the protein-nucleic acid interface; 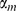 is a scaling factor which accounts for the contribution of the entropy to the specificity (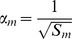, where 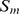 is the conformational entropy of the complex m) [19]. Here, 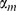 approximately depends on the number of interfacial residues/nucleotides (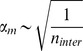) of the native protein-nucleic acid conformation of the complex m. An interfacial residue/nucleotide is defined if any atom of this residue/nucleotide in one partner of the native protein-nucleic acid conformation is within 10Å from the other partner. 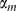 normalizes the increase of ISR with the number of interfacial residues/nucleotides. 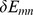 is the energy gap between the energy of a given conformation 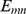 and the average energy of the conformation ensemble 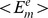 including the native conformation and all the decoys of the complex m, 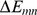 is the energy roughness or the width of the energy distribution of conformation ensemble, namely 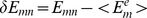 and 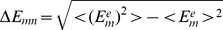, 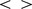 means the average over the ensemble of conformations.

### Optimization of knowledge-based statistical potentials

The iterative method [61], [65] was employed to realize the optimization. The idea of the iterative method is to circumvent the inaccessible reference state problem by adjusting the expected statistical pair potentials until they are able to discriminate native binding mode from decoys. As aforementioned, the expected potentials obtained from the ensemble of conformations generally are not equal to the potentials from the observed native conformations. The difference between the expected statistical potentials and the observed statistical potentials, as well as the iterative equation are expressed by
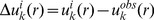
(12)

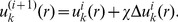
(13)



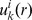
 is the expected distance-dependant potentials 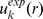 starting from i = 0 and the new expected statistical distance-dependant pair potentials 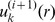 was taken to compute the 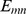 and 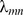, as well as 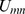 through equations (9–11). In return, the 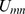 was used to update the expected pair potentials through equations (5–9). Thus, the expected pair potentials were adjusted with the difference 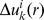 at each iteration step. The 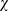 controls the speed of the convergence and was set as 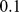. The iterative procedure was repeated until the success rate of the best-scored conformations passing the high quality accuracy of CAPRI criteria (Table S6 in File S1) converges to a high value. The resulting set of the expected pair potentials constitutes the optimized scoring function of protein-nucleic acid interactions, namely SPA-PN.

## Results and Discussion

### Optimized SPA-PN

**Figure 2:**
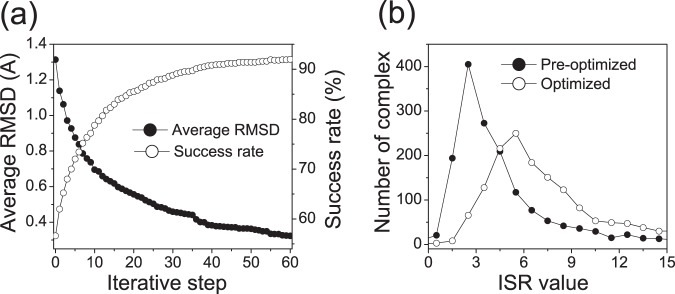
Optimization of SPA-PN. (A) Evolution of the success rate and the average interfacial RMSD (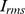) as the iteration precedes. (B) The distribution of ISR values calculated with pre-optimized SPA-PN and optimized SPA-PN respectively.

To validate the effectiveness of the iterative procedure on the improvement of the statistical potentials, we show the evolution of the average interfacial RMSD (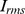, root-mean-square displacement of backbone atoms among the interface) of the best-scored poses and the success rate of the best-scored poses passing high accuracy of CAPRI evaluation criteria (Figure 2A). It can be seen that the success rate increases from 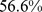 and converges to 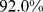 while the average 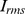 decreases from 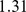 and approaches to 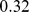 through adjusting of the atom-pair potentials via iteration. When the iteration reaches convergence, almost all the best-scored poses of the protein-nucleic acid complexes in the training set are identified as the native conformations by the optimized scoring function SPA-PN, and the structures of the best-scored poses are identical or similar to their native conformations with low 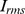
s. This suggests that the accuracy of the statistical expected knowledge-based pair potentials on the prediction of the binding affinity and identification of native conformation are improved gradually as the iteration continues until the convergence is reached. It satisfies our expectations that the optimized scoring functions is to favor the native conformations energetically as occurred in nature.

**Figure 3:**
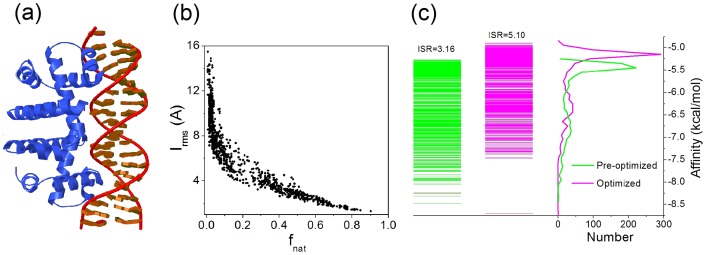
A typical example of protein-nucleic acid complex (PDB 1TRO). (A) Protein-nucleic acid binding structure with protein colored in blue and nucleic acid colored in red. (B) Plot of interfacial RMSD (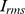) as a function of the fraction of native contacts (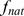) for 1000 docking decoys of the typical complex. (C) Energy spectrum and distribution calculated with pre-optimized SPA-PN (green) and optimized SPA-PN (magenta), the corresponding ISR values are shown and the energy of the native conformation is marked as red.

The novelty of our optimization strategy is to couple the optimizations of the affinity and specificity simultaneously via the iterative adjustments of the atom-pair potentials. As seen (Figure 2B), the distribution of ISR value of pre-optimized and optimized SPA-PN is clearly separated, and the average value of ISRs for the native poses increases from 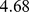 to 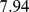. It implies that the specificity of native conformation is more pronounced while the stability is more strengthened with the optimized SPA-PN. A typical example of protein-nucleic acid docking complex (PDB ITRO) is represented with its ensemble of docking conformations (Figure 3). After optimization, the native conformation becomes more separated from the decoy ensemble while the energy distribution of decoys becomes more narrow, i.e. the energy gap between the energy of native conformation and the average energy of conformation ensemble is enlarged, while the energy roughness or the width of the energy distribution of the conformation ensemble is reduced. The ISR value of the native conformation increases from 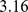 to 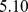 during the optimization, and also the stability of the native conformation is enhanced compared to the decoy ensemble. Collectively, the optimizing of the statistical potentials improves the performance of SPA-PN on characterizing both the stability and specificity of the native pose. A subset of atom-pair potentials of the optimized SPA-PN are shown in the Figure S2 in File S1. The potentials are normally have more than one minimum, which is consistent with the characteristic feature of the knowledge-based potential that is the mixture of different kinds of atom-pair interactions, such as electrostatic, hydrogen bonding, hydrophobic and van der Waals interactions.

Scoring functions of biomolecular recognition are generally used for two applications: (1) to predict and explain the experimentally determined affinities; and (2) to score and rank the binding poses generated by the docking programs. Thus, to validate the performance of SPA-PN, two kinds of tests related to corresponding applications are carried out and the testing results are shown in the sections below.

### Prediction of binding affinity

**Figure 4:**
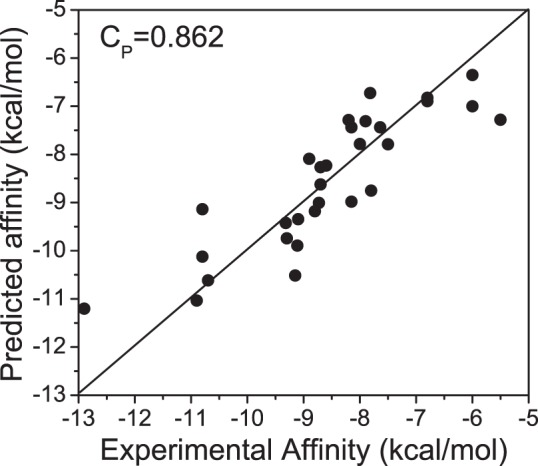
Pearson correlation between the predicted affinities calculated by SPA-PN and experimental binding affinities for 30 protein-DNA complexes of testing dataset1. The correlation coefficient (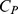) is 0.862 (statistical significance 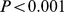). The predicted affinities are obtained by scaling the binding scores with a linear equation:y = 0.0045x-5.129 which is a fitting equation based on the experimental affinities.

The prediction accuracy of the binding affinity determines the performance of the scoring function on how well it can reproduce the experimentally measured affinity and predict the biomolecular interactions. Due to scaling, the scoring functions usually can not reproduce the absolute values of experimental binding affinity, the Pearson correlation coefficient (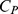) between the predicted and experimental measured binding affinities were computed for the 30 protein-DNA complexes of the testing dataset1. The 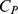
s of the scoring functions (cFIRE, DDNA, FIRE, vcFIRE and vFIRE) were obtained from the paper [44]. The correlations between the predicted and experimental affinities are shown in Table 1 and the detailed affinity values are listed in Table S2 in File S1. In order to emphasize the importance of ISR on the optimization of scoring function, we also optimized the scoring function by only taking affinity into the optimization (called as Affinity-PN), i.e. the equation 9 becomes 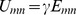. From the comparisons with other scoring functions, the performance of SPA-PN ranks best with 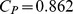 (Figure 4). The high consistence with experimental measurements indicates that SPA-PN is accurately predicting the binding affinities for the protein-nucleic acid interactions.

**Table 1:** Pearson correlations (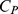) between the predicted binding affinities and experimentally measured binding affinities for 30 protein-DNA complexes of the testing dataset1.

Scoring function	*C_p_*
SPA-PN	0.862
Affinity-PN	0.857
Pre-optimized	0.817
RosettaDock	0.638
cFIRE	0.847
DDNA	0.840
FIRE	0.790
vcFIRE	0.720
vFIRE	0.550

### Identification of native pose

The aim of computational docking is to look for the native or near-native binding pose for the assembly partners. Whether the best-scored binding pose resembles the native conformation in structure determines the scoring and ranking ability of the scoring function. The performance of binding pose prediction for SPA-PN is tested on both protein-DNA and protein-RNA complexes of testing dataset2 (Table S3 in File S1). The performances of SPA-PN on identifying the native pose are compared with pre-optimized SPA-PN and affinity-PN (Table 2). The success rates of the native pose identification calculated by SPA-PN for both protein-DNA and protein-RNA complexes are over 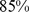 which is much higher than that of pre-optimized SPA-PN. The success rate for the testing dataset is close to that for the training dataset. This suggests that the optimized SPA-PN is successful and robust on the ability to identify native or near-native binding poses. The high success rate also means that optimized SPA-PN is effective to discriminate the native binding pose against decoys, namely able to characterize the specificity. It is worth noting that the comparison of Affinity-PN and SPA-PN demonstrates the importance of incorporation of ISR into the optimization strategy since it further improves the performance of the scoring function on the identification of native or near-native binding poses. With affinity and specificity optimization, SPA-PN outperforms Affinity-PN not only on the affinity prediction but also the identification of native conformation.

**Table 2:** Success rates (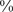) of identifying the native or near-native conformations for testing dataset2 including 232 protein-DNA and 83 protein-RNA complexes.

Scoring function	Protein-DNA	Protein-RNA	All
SPA-PN	96.6	85.5	93.7
Affinity-PN	93.5	84.3	91.1
Pre-optimized	53.0	62.7	55.6

## Conclusion

In this work, we have developed a novel scoring function SPA-PN for protein-nucleic acid interactions with the concept of intrinsic specificity. Our optimization strategy of SPA-PN satisfies the requirement that the stability of the specific complex is maximized while the stability of competing complexes is minimized. It guarantees both the stability and the specificity for the specific complex. This optimization strategy represents a significant advance over the previous investigations on protein-nucleic acid interactions which only focused on affinity. We have employed a largest set of high quality protein-nucleic acid structures so far for training the SPA-PN and includes both protein-DNA and protein-RNA complexes, making SPA-PN more independent on the training set and more generalizable for applications. The remarkable performance of SPA-PN was validated by testing the ability on the identification of native pose and prediction of binding affinity. In addition, SPA-PN is composed of statistical pair-potentials which are discrete potentials dependent on the distances between the interacting atom pairs. The statistical pair-potentials incorporate multiple energy terms into one potential energy term. Therefore, the computational docking procedure with SPA-PN will cost less time if SPA-PN is implemented into the sampling and ranking of protein-nucleic acid structure prediction.

The success of SPA-PN demonstrates that the specificity is critical to the protein-nucleic acid interactions and necessary to be incorporated into the optimization of scoring function. Similar concept was taken in computational redesign for biomolecules. Design of biomolecules requires the energy discrimination of interacting with specific partners against other competitive partners. In natural systems, evolution could encode the specificity in the functional structures against the large number of alterative ones. The quantification of the specificity for biomolecular interactions opens up a new window to explore new approaches for both the development of scoring functions and computational design of biomolecules. Our proposed quantification of specificity by ISR can be employed as a framework for further improvement of SPA-PN, and as a criteria for the computational redesign of protein-nucleic acid interface.

## Supporting Information

File S1 Supporting figures and tables. **Figure S1** The development of SPA-PN contains three stages: The preparation of database, optimization of the scoring function, Testing and application of SPA-PN. **Figure S2** Typical atom-pair interaction potentials of SPA-PN. (A and B) Two of most frequently occurred atom pairs. (C and D) Atom pairs related to hydrogen bond. (E and F) Atom pairs involving phosphorus atom. **Table S1** Training dataset for the development of SPA-PN. **Table S2** Experimental determined affinities and SPA-PN predicted affinities for 30 protein-DNA complexes of the testing dataset1, the calculated affinities were obtained by scaling the binding scores with linear fitting equations (SPA-PN: y = 0.0045*x-5.129, Affinity-PN:y = 0.0044x-5.080, Pre-optimized: 0.0043x-5.443, Rosettadock: y = 0.0053x-7.24) based on the experimental affinities. **Table S3** PDB codes of the testing dataset2. **Table S4** 15 Atom types used for calculating the atom pair potentials based on the SYBYL definition of atom type. The atom types can be converted from PDB files by the software OpenBabel. **Table S5** 95 effective types of atom pairs for the protein-nucleic acid interactions with the cutoff of total occurrences larger than 600 in the training dataset. **Table S6** The high accuracy quality of CAPRI assessment criteria was taken to define the near-native conformation.
(DOC)
