# Supplementary material for: Optimizing Scoring Function of Protein-Nucleic Acid Interactions with Both Affinity and Specificity
Source: PLoS One. 2013 Sep 30;8(9):e74443. doi: 10.1371/journal.pone.0074443 (PMC3787031; doi:10.1371/journal.pone.0074443)
Supplement: File S1 — Supporting figures and tables. Figure S1 The development of SPA-PN contains three stages: The preparation of database, optimization of the scoring function, Testing and application of SPA-PN. Figure S2 Typical atom-pair interaction potentials of SPA-PN. (A and B) Two of most frequently occurred atom pairs. (C and D) Atom pairs related to hydrogen bond. (E and F) Atom pairs involving phosphorus atom. Table S1 Training dataset for the development of SPA-PN. Table S2 Experimental determined affinities and SPA-PN predicted affinities for 30 protein-DNA complexes of the testing dataset1, the calculated affinities were obtained by scaling the binding scores with linear fitting equations (SPA-PN: y = 0.0045*x-5.129, Affinity-PN:y = 0.0044x-5.080, Pre-optimized: 0.0043x-5.443, Rosettadock: y = 0.0053x-7.24) based on the experimental affinities. Table S3 PDB codes of the testing dataset2. Table S4 15 Atom types used for calculating the atom pair potentials based on the SYBYL definition of atom type. The atom types can be converted from PDB files by the software OpenBabel. Table S5 95 effective types of atom pairs for the protein-nucleic acid interactions with the cutoff of total occurrences larger than 600 in the training dataset. Table S6 The high accuracy quality of CAPRI assessment criteria was taken to define the near-native conformation. (DOC) [file pone.0074443.s001.doc]

**Supporting Information**

**Figure S1** The development of SPA-PN contains three stages: The preparation of database, optimization of the scoring function, Testing and application of SPA-PN.


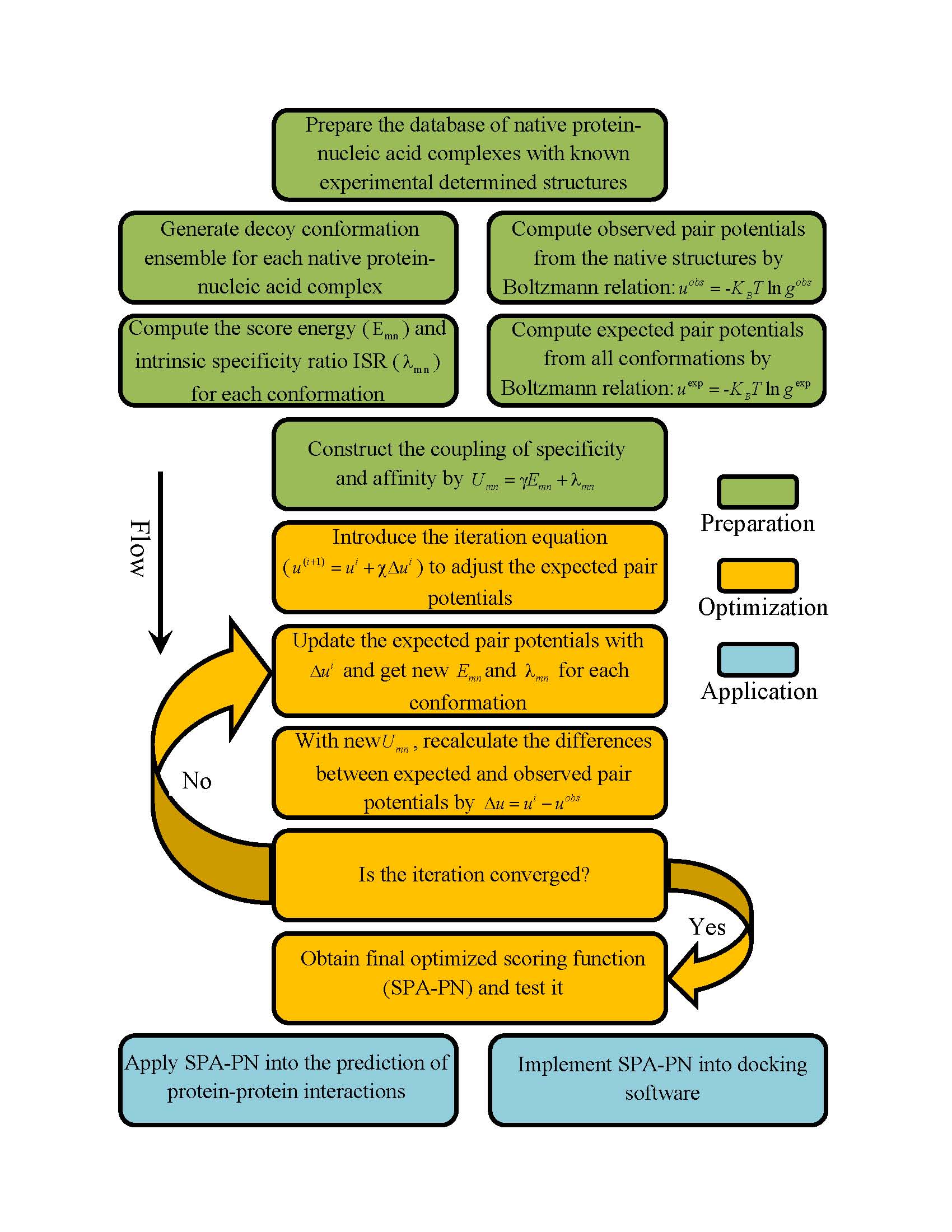


**Figure S2** Typical atom-pair interaction potentials of SPA-PN. (A and B) Two of most frequently occurred atom pairs. (C and D) Atom pairs related to hydrogen bond. (E and F) Atom pairs involving phosphorus atom.


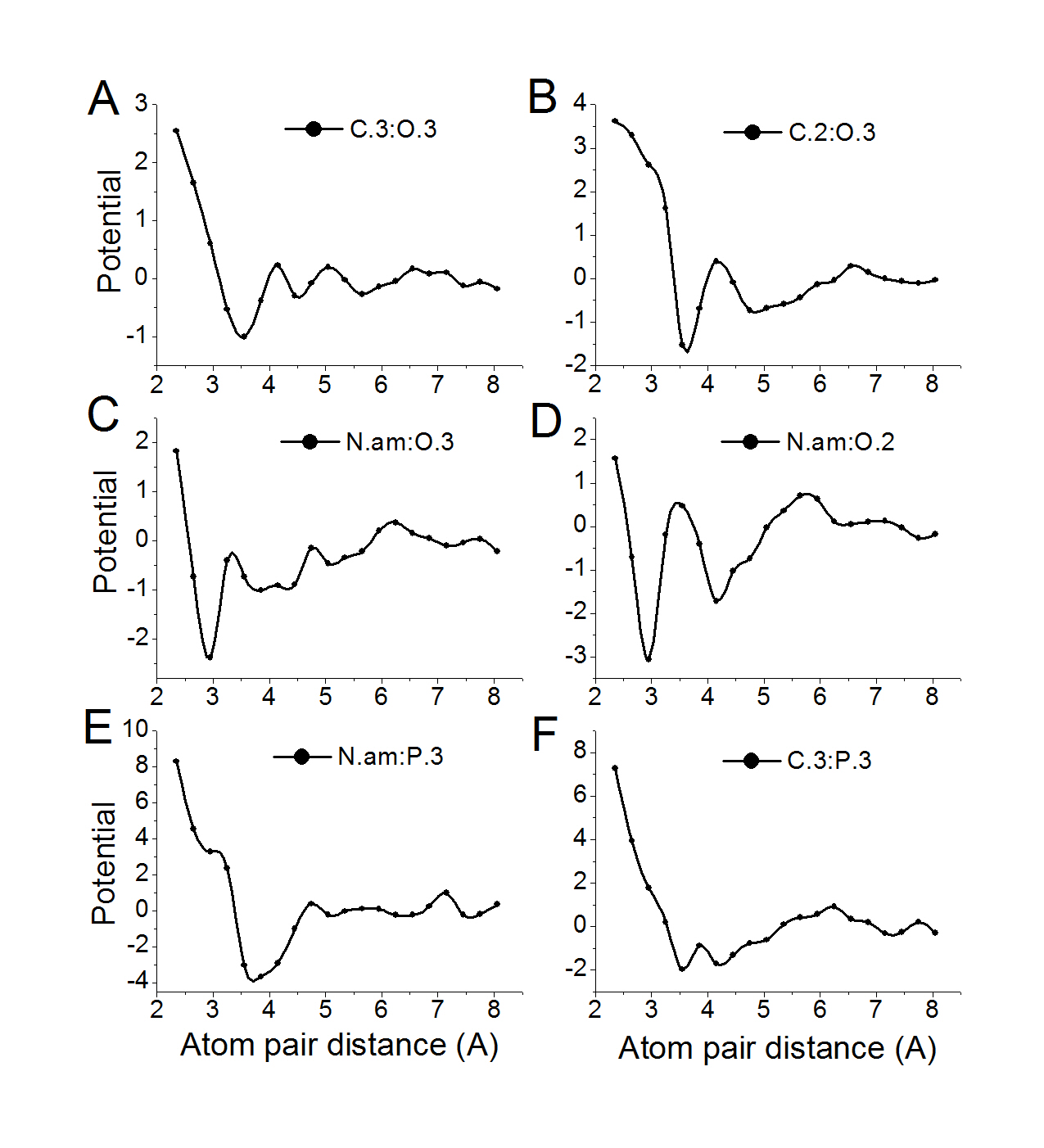


| **Table S1** Training dataset for the development of SPA-PN.  **PDB codes of 1221 protein-DNA complexes in the training dataset** |
| --- |
| 10MH 1A02 1A0A 1A1F 1A1G 1A1H 1A1I 1A1J 1A1K 1A1L 1A31 1AAY 1AN2  1AN4 1APL 1AZ0 1AZQ 1B95 1B96 1B97 1BC7 1BC8 1BDH 1BDI 1BDV 1BF4  1BF5 1BGB 1BHM 1BNK 1BNZ 1BP7 1BPY 1BPZ 1BSS 1BSU 1BUA 1BVO 1C8C  1C9B 1CA5 1CA6 1CDW 1CF7 1CIT 1CL8 1CLQ 1CYQ 1CZ0 1D0E 1D1U 1D5Y  1D66 1DCT 1DE8 1DEW 1DGC 1DH3 1DNK 1DU0 1E3M 1EBM 1ECR 1EFA 1EMJ  1EO3 1EO4 1EON 1EOO 1EOP 1ERI 1EWN 1EXJ 1EYG 1F0O 1F4R 1F5T 1FJX  1FN7 1FZP 1G2D 1G2F 1G38 1GA5 1GJI 1GLU 1GT0 1H0M 1H88 1H8A 1H9D  1HAO 1HAP 1HDD 1HF0 1HJB 1HLO 1HLZ 1HUO 1HUT 1HUZ 1I6J 1I7D 1IC8  1IF1 1IG9 1IJW 1IMH 1IO4 1IPP 1JB7 1JFI 1JFS 1JFT 1JH9 1JJ6 1JJ8  1JK1 1JK2 1JKO 1JKP 1JKQ 1JKR 1JMC 1JNM 1JX4 1JXL 1K3X 1K7A 1KB2  1KB4 1KB6 1KFV 1KIX 1L1T 1L1Z 1L2C 1L2D 1L3S 1L3T 1L3U 1L3V 1L5U  1LAT 1LE5 1LE9 1LEI 1LLI 1LRR 1LV5 1LWV 1LWW 1LWY 1M3H 1M3Q 1M5R  1MDM 1MDY 1MHD 1MHT 1MJM 1MJO 1MJQ 1MQ3 1MTL 1MW8 1MWI 1MWJ 1N39  1N3A 1N3C 1N56 1N6Q 1NG9 1NGM 1NJW 1NJX 1NJY 1NJZ 1NK0 1NK4 1NK5  1NK6 1NK7 1NK8 1NK9 1NKB 1NKC 1NKE 1NNJ 1NWQ 1OCT 1ODG 1ODH 1OE5  1OE6 1OH5 1OH6 1OH8 1OMH 1ORP 1OSB 1OTC 1OUZ 1OWF 1OWG 1OWR 1P51  1P59 1P78 1P7D 1P7H 1PA6 1PAR 1PGZ 1PH1 1PH3 1PH4 1PH5 1PH6 1PH7  1PH8 1PH9 1PHJ 1PJI 1PJJ 1PM5 1PNR 1PO6 1PVI 1Q3F 1Q9X 1Q9Y 1QAI  1QBJ 1QN3 1QN4 1QN5 1QN6 1QN7 1QN8 1QN9 1QNA 1QNB 1QNC 1QP0 1QP4  1QP7 1QP9 1QQA 1QQB 1QRH 1QRI 1QSS 1QSY 1QX0 1QZG 1QZH 1R0A 1R0N  1R2Y 1R2Z 1R4R 1R8D 1RAM 1REP 1RRQ 1RV5 1RVB 1RYR 1RYS 1RZR 1S0M  1S0N 1S0O 1S10 1S6M 1S97 1S9F 1SAX 1SKM 1SKS 1SKW 1SL1 1SL2 1SSP  1SUZ 1SVC 1SX8 1SXP 1SXQ 1T05 1T2K 1T2T 1T3N 1T7P 1T8E 1T8I 1T9I  1T9J 1TAU 1TGH 1TK0 1TK5 1TK8 1TQE 1TSR 1TTU 1TUP 1TV9 1TVA 1TW8  1TX3 1U0C 1U0D 1U1K 1U1L 1U1M 1U1N 1U1O 1U1P 1U1Q 1U1R 1U3E 1U45  1U47 1U48 1U49 1U4B 1U78 1U8R 1UA0 1UA1 1UAA 1UUT 1V14 1VKX 1VOL  1VPW 1VRL 1VTL 1VTN 1VTO 1WB9 1WBB 1WBD 1WD0 1WD1 1WET 1WTO 1WTQ  1WTR 1WTV 1WTW 1WTX 1X9M 1X9N 1X9S 1X9W 1XC8 1XC9 1XJV 1XPX 1XSD  1XYI 1Y6G 1Y8Z 1YA6 1YFI 1YFJ 1YNW 1YQK 1YQL 1YQM 1YQR 1YRN 1YSA  1YTB 1YTF 1ZAA 1ZAY 1ZET 1ZG1 1ZG5 1ZJM 1ZJN 1ZM5 1ZNS 1ZQI 1ZQN  1ZTG 1ZVV 1ZX4 1ZYQ 1ZZI 1ZZJ 2A0I 2A6O 2ACJ 2AGO 2AGP 2AGQ 2AJQ  2AOQ 2AS5 2ASD 2ASJ 2ASL 2ATL 2AU0 2B0D 2B0E 2BAM 2BDP 2BGW 2BPF  2BQ3 2BQR 2BQU 2BR0 2BSQ 2C22 2C28 2C2D 2C2E 2C2R 2C62 2C7A 2C7O  2C7P 2C7Q 2C7R 2CCZ 2CDM 2DEM 2DP6 2DRP 2DY4 2E42 2E43 2EA0 2EFW  2ER8 2ERE 2ES2 2EUV 2EUW 2EUX 2EUZ 2EVF 2EVG 2EVH 2EVI 2EVJ 2EWJ  2F5N 2F5O 2F5P 2F5Q 2F5S 2FLD 2FMP 2FMQ 2FMS 2FR4 2FVP 2FVQ 2FVR  2FVS 2GB7 2GEQ 2GIE 2GIG 2GII 2GIJ 2GLI 2H1K 2H1O 2H7F 2HAP 2HAX  2HEO 2HHQ 2HHS 2HHT 2HHU 2HHV 2HHW 2HMI 2HOS 2HOT 2HR1 2HVH 2HVI  2HW3 2I05 2I0Q 2I3P 2I3Q 2I5W 2I9G 2I9K 2I9T 2IBK 2IBS 2IBT 2IEF  2IH2 2IH4 2IH5 2IHN 2IIF 2IS1 2IS2 2IS4 2ISO 2ISP 2IT0 2IVH 2J6S  2J6T 2J6U 2JEF 2JEG 2JEI 2JEJ 2JG3 2KTQ 2NL8 2NNY 2NOB 2NOE 2NOF  2NOH 2NOI 2NOL 2NOZ 2NP7 2NQJ 2O19 2O4A 2O54 2O59 2O5C 2O5E 2O61  2O6M 2O8B 2O8D 2OG0 2OPF 2OQ4 2OR1 2OXM 2OXV 2OYT 2OZM 2OZS 2P2R  2P5G 2P5L 2P5O 2P66 2P6R 2PI4 2PI5 2PQU 2PUA 2PUB 2PUC 2PUD 2PUE  2PUF 2PUG 2PVI 2PXI 2PY5 2PYL 2PZS 2Q10 2Q2K 2Q2T 2Q2U 2QSH 2R5Y  2R5Z 2R8G 2R8H 2R8I 2RBA 2RDJ 2RVE 2SSP 2UP1 2UVR 2UVU 2UVW 2UYC  2UYH 2UZ4 2UZK 2V4Q 2V4R 2V9W 2VA2 2VA3 2VBJ 2VBO 2VHG 2VIC 2VIH  2VJU 2VW9 2VWJ 2VY2 2VZ4 2W42 2W8L 2W9A 2W9C 2WB2 2WBS 2WBU 2WIW  2WQ6 2WQ7 2WT7 2WTF 2WTY 2X6V 2XC9 2XCA 2XCP 2XCS 2XE0 2XGQ 2XHB  2XHI 2XO7 2XRZ 2XSD 2XY5 2XY6 2XZF 2XZU 2Y1I 2Y1J 2Z6A 2Z6Q 2Z6U  2ZCJ 2ZHG 2ZKD 2ZKE 2ZKF 2ZO0 3A01 3A46 3A5T 3A5U 3AAF 3AUO 3B39  3BDP 3BI3 3BJY 3BKZ 3BQ0 3BQ1 3BQ2 3BTY 3BTZ 3BU0 3BUC 3C0W 3C0X  3C2K 3C2L 3C2M 3C2P 3C3L 3C46 3C58 3CFP 3CFR 3CLC 3CLZ 3CMW 3CO7  3COA 3CQ8 3CVV 3CVY 3D0A 3D0P 3D1N 3D2W 3D6Y 3D6Z 3D70 3D71 3DFX  3DLH 3DNV 3DSC 3DSD 3DVO 3DW9 3E3Y 3E40 3E41 3E42 3E43 3E45 3EBC  3EEO 3EH8 3EI1 3EI2 3EY1 3EYI 3EYZ 3EZ5 3F27 3F2B 3F2C 3F2D 3F8I  3F8J 3FBD 3FC3 3FD2 3FDE 3FDQ 3FMT 3FSP 3FSQ 3FYL 3G0Q 3G6P 3G6R  3G6T 3G6U 3G6V 3G6X 3G6Y 3G73 3G8U 3G8X 3G97 3G99 3G9J 3G9M 3G9O  3G9P 3GDX 3GII 3GIJ 3GIK 3GIL 3GIM 3GO8 3GOX 3GP1 3GP8 3GPL 3GPP  3GPU 3GPX 3GPY 3GQ3 3GQ4 3GQ5 3GQC 3GV5 3GV7 3GV8 3GX4 3GXQ 3GYH  3GZ6 3H0D 3H25 3H40 3H4B 3H4D 3H8O 3H8R 3H8X 3HDD 3HP6 3HPO 3HQF  3HT3 3HTS 3HXO 3HXQ 3HZI 3I0W 3I0X 3I8D 3IAG 3IAY 3IGK 3IGL 3IKT  3IL2 3IMB 3IRQ 3IRR 3ISB 3ISC 3ISD 3IV5 3JPN 3JPO 3JPP 3JPQ 3JPR  3JPS 3JPT 3JR4 3JR5 3JR9 3JRH 3JSM 3JSO 3JSP 3JTG 3JX7 3JXB 3JXC  3JXD 3JXY 3JXZ 3JY1 3K0S 3K4X 3K57 3K58 3K59 3K5L 3K5M 3KD1 3KD5  3KDE 3KHG 3KHH 3KHL 3KHR 3KJP 3KK1 3KK2 3KK3 3KLH 3KMD 3KMP 3KO2  3KOV 3KQH 3KQK 3KQL 3KQN 3KQU 3KTQ 3KTU 3KXT 3KZ8 3L1P 3L2C 3L2P  3L4J 3L4K 3L8B 3LDS 3LK9 3LNQ 3LSP 3LSR 3LWH 3LWI 3LWL 3LWM 3LZI  3LZJ 3M4A 3M8R 3M8S 3M9E 3M9M 3M9O 3MAQ 3MBY 3MFH 3MFI 3MFK 3MHT  3MIP 3MIS 3MKW 3MKY 3MKZ 3MLN 3MLP 3MQ6 3MR2 3MR3 3MR5 3MR6 3MU6  3MVA 3MVB 3MX4 3MX9 3MXB 3MZH 3N78 3N7B 3N7Q 3NAE 3NCI 3NDH 3NDK  3NE6 3NGD 3NGI 3NHG 3NIC 3O1M 3O1O 3O1P 3O1R 3O1S 3O1T 3O1U 3O1V  3O9X 3OD8 3ODA 3ODC 3ODE 3ODH 3OGD 3OGU 3OH6 3OH9 3OHA 3OHB 3OJS  3OJU 3ON0 3OOL 3OOR 3OQG 3OQM 3OQO 3ORC 3OSF 3OSG 3OSN 3OSP 3PO4  3PO5 3POV 3PR4 3PR5 3PT6 3PV8 3PVI 3PVP 3PVV 3PW0 3PW2 3PW4 3PW5  3PW7 3PX0 3PX4 3PX6 3PY8 3Q05 3Q0A 3Q0B 3Q0C 3Q0D 3Q0F 3Q22 3Q23  3Q24 3Q2Y 3Q3D 3Q5F 3Q5P 3Q8K 3Q8L 3Q8M 3Q8P 3Q8R 3Q8S 3QE9 3QEB  3QEI 3QEP 3QER 3QES 3QET 3QEV 3QEW 3QEX 3QFQ 3QI5 3QLP 3QMB 3QMC  3QMD 3QMG 3QMH 3QMI 3QNN 3QNO 3QQY 3QRF 3QSV 3QWS 3QYN 3QZ7 3QZ8  3RAQ 3RAX 3RB3 3RB4 3RB6 3RBD 3RBE 3RH4 3RH5 3RH6 3RJE 3RJF 3RJG  3RJH 3RJI 3RJJ 3RJK 3RKQ 3RMB 3RMC 3RN2 3RNU 3RR7 3RR8 3RRG 3RRH  3RTV 3RWU 3RZG 3RZH 3RZJ 3RZK 3RZL 3S57 3S59 3S5A 3S6I 3S8Q 3S9H  3SAR 3SAS 3SAT 3SAU 3SAV 3SAW 3SBJ 3SCX 3SI6 3SJJ 3SLP 3SM4 3SNN  3SPD 3SPL 3SPY 3SPZ 3SQ0 3SQ1 3SQ2 3SQ4 3SQI 3SSC 3SSD 3SSE 3SUN  3SUO 3SUP 3SV3 3SV4 3SYZ 3SZ2 3T3F 3T5H 3T5J 3T5K 3T5L 3TAB 3TAE  3TAF 3TAG 3TAN 3TAP 3TAQ 3TAR 3TED 3TFR 3TFS 3THV 3THX 3THY 3TI0  3TMM 3TQ1 3TQ6 3TS8 3U2B 3U4Q 3U6L 3UBY 3UFJ 3UGM 3UGO 3UGP 3UIQ  3UKG 3ULP 3UO7 3US0 3US1 3UVF 3V1Z 3V20 3V4I 3V6D 3V6J 3V6T 3V81  3VK7 3VK8 3VKE 3ZQC 3ZQL 3ZVK 4A04 4A08 4AA6 4AAE 4AAF 4AAG 4AIL  4BDP 4DA4 4DF4 4DF8 4DFJ 4DFK 4DFM 4DFP 4DK9 4DL2 4DL3 4DL4 4DL5  4DL6 4DL7 4DO9 4DOA 4DOB 4DOC 4DQI 4DQP 4DQQ 4DQR 4DS4 4DS5 4DSE  4DSF 4DTJ 4DTM 4DTN 4DTO 4DTP 4DTR 4DTS 4DTU 4DTX 4DU1 4DU3 4DU4  4E0D 4E3S 4EBC 4EBD 4EBE 4EFJ 4ENM 4ENN 4ESJ 4EUW 4F41 4F43 4MHT  4RVE 4SKN 5MHT 6MHT 6PAX 7ICM 7ICP 7ICQ 7ICR 7ICT 8ICC 8ICK 8ICN  8ICO 8ICP 8ICQ 8ICR 8ICS 8ICX 8MHT 9ICF 9ICH 9ICM 9ICN 9ICQ 9ICR  9ICS 9ICT 9ICV 9MHT |
| **PDB codes of 334 protein-RNA complexes in the training dataset** |
| 1A9N 1ASZ 1AV6 1CVJ 1CX0 1DI2 1DRZ 1DUL 1EUQ 1EUY 1EXD 1F7V 1F7Y  1FFY 1G2E 1G59 1GAX 1GTR 1GTS 1H4Q 1I6U 1IL2 1IVS 1JBT 1JID 1KNZ  1KUQ 1M5K 1M5P 1M5V 1M8X 1M8Y 1MZP 1N77 1O0B 1O0C 1OB5 1P6V 1Q2S  1QA6 1QRS 1QRT 1QRU 1QU2 1QU3 1SI3 1SJ3 1SJ4 1SJF 1UN6 1URN 1VBX  1VBY 1VBZ 1VC0 1VC5 1VC6 1VC7 1WZ2 1XOK 1Y39 1YTY 1ZE2 1ZH5 1ZHO  1ZJW 1ZL3 2AB4 2AKE 2ANN 2ANR 2ATW 2AZ2 2BYT 2CT8 2CV0 2CV1 2CV2  2DET 2DEU 2DR2 2DR5 2DR7 2DR8 2DR9 2DVI 2DXI 2E9R 2E9T 2E9Z 2EC0  2F8T 2G4B 2HVY 2HYI 2J0Q 2J0S 2JLU 2JLV 2JLW 2JLX 2JLY 2JLZ 2NUE  2NUF 2NZ4 2OIH 2OJ3 2OZB 2PJP 2PLY 2PXB 2PXD 2PXE 2PXF 2PXK 2PXL  2PXP 2PXQ 2PXT 2PXU 2PXV 2R7S 2R7T 2R7U 2R7V 2R7W 2R7X 2RD2 2RE8  2RFK 2UWM 2V0G 2VNU 2VOD 2VON 2VPL 2W2H 2XB2 2XD0 2XDB 2XDD 2XGJ  2XS2 2XS5 2XZO 2Y8W 2Y8Y 2Y9H 2YJY 2YKG 2ZH1 2ZH2 2ZH3 2ZH4 2ZH5  2ZH6 2ZH7 2ZH8 2ZH9 2ZHA 2ZHB 2ZI0 2ZM5 2ZNI 2ZXU 2ZZM 2ZZN 3ADB  3ADC 3ADD 3ADI 3ADL 3AEV 3AHU 3AKZ 3AL0 3AM1 3AMT 3AMU 3AVU 3AVV  3AVW 3AVX 3AVY 3BO3 3BOY 3BSN 3BX3 3CUL 3CUN 3DH3 3EPH 3EPJ 3EPK  3EQT 3EX7 3FHT 3FOZ 3FTE 3FTF 3G0H 3G8S 3G8T 3G9C 3G9Y 3GIB 3H5X  3H5Y 3HAX 3HHN 3HJW 3HL2 3HSB 3HTX 3I5X 3I5Y 3I61 3I62 3IAB 3IEV  3IRW 3IVK 3IWN 3K0J 3K49 3K4E 3K5Q 3K5Y 3K5Z 3K61 3K62 3K64 3KLV  3KMQ 3KMS 3KNA 3KS8 3KTW 3L25 3L26 3L3C 3LQX 3LRN 3LRR 3LWO 3LWP  3LWQ 3LWR 3LWV 3MDG 3MDI 3MJ0 3MOJ 3MQK 3MUM 3MUR 3MUT 3MUV 3MXH  3NCU 3NDB 3NL0 3NMR 3NMU 3NNA 3NNC 3NNH 3O3I 3O6E 3O7V 3O8C 3O8R  3OG8 3OIJ 3OIN 3OUY 3OV7 3OVA 3OVB 3OVS 3PEW 3PEY 3PKM 3Q0L 3Q0M  3Q0N 3Q0O 3Q0P 3Q0Q 3Q0R 3Q0S 3Q2T 3QG9 3QGB 3QGC 3QJJ 3QJL 3QJP  3QRP 3QRR 3QSY 3R1H 3R1L 3R2C 3R2D 3R9W 3R9X 3RC8 3RW6 3SIU 3SIV  3SN2 3SQW 3SQX 3T5N 3T5Q 3TMI 3TRZ 3TS0 3TS2 3TUP 3V6Y 3V71 3V74  3V7E 4AL5 4AL6 4AL7 4ARI 4AS1 4DZS 4ERD 4F1N |

**Table S2** Experimental determined affinities and SPA-PN predicted affinities for 30 protein-DNA complexes of the testing dataset1, the calculated affinities were obtained by scaling the binding scores with linear fitting equations (SPA-PN: y=0.0045*x-5.129, Affinity-PN:y=0.0044x-5.080, Pre-optimized: 0.0043x-5.443, Rosettadock: y=0.0053x-7.24) based on the experimental affinities.

| **PDB code** | **Chain ID** | **Experimental**  **(kcal/mol)** | **SPA-PN (kcal/mol)** | **Affinity-PN**  **(kcal/mol)** | **Pre-optimized**  **(kcal/mol)** | **RosettaDock (kcal/mol)** |
| --- | --- | --- | --- | --- | --- | --- |
| 1AAY | A/BC | -8.90 | -8.09 | -8.36 | -8.463 | -7.720 |
| 1APL | AB/D | -7.82 | -6.726 | -6.736 | -6.911 | -7.559 |
| 1AZ0 | AB/CD | -12.90 | -11.205 | -11.161 | -10.737 | -10.268 |
| 1AZP | A/BC | -6.80 | -6.895 | -7.017 | -6.949 | -7.645 |
| 1BC7 | AB/C | -7.90 | -7.31 | -7.399 | -7.556 | -7.842 |
| 1BHM | AB/CD | -9.11 | -9.894 | -9.969 | -9.625 | -9.811 |
| 1BP7 | B/12 | -8.15 | -8.982 | -8.932 | -9.012 | -8.189 |
| 1CA5 | A/BC | -6.00 | -7.000 | -7.104 | -6.904 | -7.602 |
| 1CDW | A/BC | -9.32 | -9.425 | -9.432 | -9.562 | -8.401 |
| 1CMA | AB/CD | -5.50 | -7.282 | -7.262 | -7.276 | -8.446 |
| 1CW0 | A/MNO | -9.10 | -9.344 | -9.403 | -9.193 | -8.213 |
| 1ECR | A/BC | -10.70 | -10.613 | -10.689 | -10.925 | -9.074 |
| 1EFA | AB/CD | -10.80 | -10.125 | -10.208 | -9.59 | -11.29 |
| 1GLU | B/DC | -6.00 | -6.354 | -6.326 | -6.613 | -7.618 |
| 1HCQ | AB/CD | -8.70 | -8.266 | -8.304 | -8.353 | -8.043 |
| 1HCR | A/BC | -8.00 | -7.782 | -7.801 | -7.223 | -7.577 |
| 1IHF | AB/CDE | -9.30 | -9.743 | -9.85 | -9.868 | -8.424 |
| 1IPP | AB/CD | -9.15 | -10.514 | -10.617 | -10.942 | -9.106 |
| 1LMB | 12/34 | -8.73 | -9.007 | -8.963 | -8.903 | -8.396 |
| 1MDY | AB/EF | -8.70 | -8.623 | -8.54 | -8.246 | -7.98 |
| 1NFK | AB/CD | -10.80 | -9.136 | -9.283 | -8.913 | -10.697 |
| 1OCT | AB/C | -7.80 | -8.751 | -8.817 | -8.66 | -8.049 |
| 1PAR | ABCD/EF | -10.90 | -11.034 | -11.3 | -9.913 | -8.561 |
| 1PUE | AB/E | -7.64 | -7.436 | -7.445 | -7.493 | -7.783 |
| 1QRV | A/CD | -8.2 | -7.286 | -7.292 | -7.489 | -7.674 |
| 1RUN | A/CF | -6.8 | -6.825 | -6.89 | -6.776 | -8.387 |
| 1TRO | AC/IJ | -8.6 | -8.231 | -8.189 | -8.151 | -8.573 |
| 1TSR | ABC/EF | -8.15 | -7.436 | -7.444 | -7.416 | -10.357 |
| 1YSA | AB/CD | -7.5 | -7.792 | -7.829 | -8.156 | -7.908 |
| 1YTF | ABCD/EF | -8.8 | -9.181 | -9.225 | -9.535 | -9.585 |

**Table S3** PDB codes of the testing dataset2.

| **PDB codes of 232 protein-DNA complexes in the testing dataset2** |
| --- |
| 1A35 1A3Q 1A6Y 1A74 1AIS 1AKH 1AU7 1AWC 1AZP 1B3T 1B72 1B8I 1B94  1BDT 1BG1 1BL0 1BPX 1BY4 1CEZ 1CKQ 1CKT 1CMA 1D02 1D2I 1D3U 1DC1  1DDN 1DFM 1DIZ 1DMU 1DP7 1DSZ 1E3O 1EA4 1EGW 1EMH 1ESG 1EWQ 1EYU  1F2I 1F4K 1F6O 1FJL 1FOK 1G9Y 1G9Z 1GD2 1GXP 1H6F 1H89 1H9T 1HCQ  1HCR 1HJC 1HLV 1HU0 1HWT 1I3J 1IG7 1IGN 1IHF 1IXY 1J1V 1J3E 1J75  1JE8 1JEY 1JGG 1JJ4 1JT0 1K3W 1K61 1K78 1K79 1K82 1KC6 1KSY 1KU7  1L3L 1LE8 1LLM 1LMB 1LQ1 1M0E 1M5X 1MEY 1MJ2 1MNM 1MNN 1MOW 1N48  1N6J 1NFK 1NH2 1NKP 1NLW 1NVP 1O3T 1OE4 1ORN 1OUP 1OZJ 1P47 1P71  1PDN 1PER 1PP7 1PUE 1PUF 1QAJ 1QNE 1QPZ 1QRV 1QTM 1QUM 1R0O 1R4O  1R7M 1R8E 1RH6 1RIO 1RM1 1RPE 1RRS 1RVA 1RXW 1SA3 1SFU 1SKN 1SKR  1TC3 1TDZ 1TEZ 1TKD 1TRO 1TRR 1UBD 1V15 1VAS 1VRR 1W0T 1W0U 1W7A  1WTE 1XBR 1Y6F 1YF3 1YO5 1Z63 1Z9C 1ZME 1ZS4 2A66 2AOR 2AQ4 2B9S  2BOP 2C5R 2C6Y 2C9L 2D5V 2DDG 2DGC 2DTU 2E52 2ETW 2EX5 2FCC 2FIO  2FKC 2FL3 2FQZ 2G1P 2GE5 2GIH 2H27 2H7G 2H7H 2HAN 2HDD 2HHX 2I06  2I13 2IIE 2IRF 2IS6 2ISZ 2NLL 2NP6 2NTC 2O49 2OAA 2ODI 2OFI 2OWO  2P0J 2PI0 2PYJ 2QHB 2QL2 2QOJ 2R1J 2R9L 2RAM 2RBF 2VE9 2VLA 2VOA  2VS7 2VY1 2Z3X 2ZO1 3BAM 3BEP 3BIE 3BM3 3BRD 3BRF 3BRG 3BS1 3BTX  3C25 3C2I 3CBB 3CO6 3COQ 3CRO 3CVU 3DPG 4KTQ 7MHT 9ANT |
| **PDB codes of 83 protein-RNA complexes in the testing dataset2** |
| 1ASY 1B7F 1C0A 1DFU 1DK1 1E7K 1E8O 1EC6 1EFW 1F7U 1FEU 1FXL 1G1X  1H3E 1H4S 1HC8 1HQ1 1J1U 1JBR 1JBS 1K8W 1KOG 1KQ2 1LNG 1M5O 1MFQ  1MJI 1MMS 1N35 1N78 1OB2 1OOA 1Q2R 1QF6 1QTQ 1R3E 1RLG 1S03 1SDS  1T0K 1TTT 1U0B 1U63 1VFG 1WNE 1WSU 1YVP 1ZBH 2ASB 2AZ0 2AZX 2B3J  2BGG 2BH2 2BTE 2CSX 2CZJ 2D6F 2DB3 2DER 2DLC 2DRA 2DRB 2EZ6 2F8K  2F8S 2FMT 2GJE 2HW8 2I82 2I91 2IX1 2PY9 2QUX 2R7R 2R8S 2V3C 2ZKO  3BSB 3BSO 3BSX 3BX2 3CIY |
|  |

**Table S4** 15 Atom types used for calculating the atom pair potentials based on the SYBYL definition of atom type. The atom types can be converted from PDB files by the software OpenBabel.

| **Type** | **Description** |
| --- | --- |
| C.3 | carbon sp3 |
| C.2 | carbon sp2 |
| C.ar | carbon aromatic |
| C.cat | other carbons |
| N.ar | nitrogen aromatic |
| N.p13 | nitrogen trigonal planar |
| N.am | nitrogen amide |
| N.2 | nitrogen sp2 |
| N.3 | nitrogen sp3 |
| N.4 | quaternary nitrogen |
| O.3 | oxygen sp3 |
| O.2 | oxygen sp2 |
| O.co2 | carboxyl oxygen |
| S.3 | sulfur sp3 |
| P.3 | phosphorous sp3 |

**Table S5** 95 effective types of atom pairs for the protein-nucleic acid interactions with the cutoff of total occurrences larger than 600 in the training dataset.

| **Atom pair** | **Atom pair** | **Atom pair** |
| --- | --- | --- |
| C.3:C.3 | C.ar:N.ar | N.ar:O.2 |
| C.3:C.2 | C.ar:N.am | N.ar:O.co2 |
| C.3:C.ar | C.ar:N.pl3 | N.ar:S.3 |
| C.3:C.cat | C.ar:N.4 | N.ar:P.3 |
| C.3:N.3 | C.ar:O.3 | N.am:N.am |
| C.3:N.2 | C.ar:O.2 | N.am:N.pl3 |
| C.3:N.ar | C.ar:O.co2 | N.am:N.4 |
| C.3:N.am | C.ar:S.3 | N.am:O.3 |
| C.3:N.pl3 | C.ar:P.3 | N.am:O.2 |
| C.3:N.4 | C.cat:N.ar | N.am:P.3 |
| C.3:O.3 | C.cat:N.pl3 | N.pl3:N.pl3 |
| C.3:O.2 | C.cat:O.3 | N.pl3:N.4 |
| C.3:O.co2 | C.cat:O.2 | N.pl3:O.3 |
| C.3:S.3 | C.cat:P.3 | N.pl3:O.2 |
| C.3:P.3 | N.3:N.3 | N.pl3:O.co2 |
| C.2:C.2 | N.3:N.2 | N.pl3:S.3 |
| C.2:C.ar | N.3:N.ar | N.pl3:P.3 |
| C.2:C.cat | N.3:N.am | N.4:O.3 |
| C.2:N.3 | N.3:N.pl3 | N.4:O.2 |
| C.2:N.2 | N.3:O.3 | N.4:P.3 |
| C.2:N.ar | N.3:O.2 | O.3:O.3 |
| C.2:N.am | N.3:P.3 | O.3:O.2 |
| C.2:N.pl3 | N.2:N.ar | O.3:O.co2 |
| C.2:N.4 | N.2:N.am | O.3:S.3 |
| C.2:O.3 | N.2:N.pl3 | O.3:P.3 |
| C.2:O.2 | N.2:O.3 | O.2:O.2 |
| C.2:O.co2 | N.2:O.2 | O.2:O.co2 |
| C.2:P.3 | N.ar:N.ar | O.2:S.3 |
| C.ar:C.ar | N.ar:N.am | O.2:P.3 |
| C.ar:C.cat | N.ar:N.pl3 | O.co2:P.3 |
| C.ar:N.3 | N.ar:N.4 | S.3:P.3 |
| C.ar:N.2 | N.ar:O.3 |  |

**Table S6** The high accuracy quality of CAPRI assessment criteria was taken to define the near-native conformation.

CAPRI assessment criteria

| **Quality** | **Combination of three parameters^a^ (f_nat_, L_rms_ and I_rms_)** |
| --- | --- |
| High accuracy | f_nat_≥0.5 and (L_rms_1.0 or I_rms_1.0) |
| Medium accuracy | (0.3f_nat_<0.5) and (L_rms_5.0 or I_rms_2.0) or (f_nat_≥0.5 and L_rms_>1.0 and I_rms_>1.0) |
| Acceptable accuracy | (0.1f_nat_<0.3) and (L_rms_10.0 or I_rms_4.0) or (f_nat_≥0.3 and L_rms_>5.0 and I_rms_>2.0) |
| Incorrect | F_nat_<0.1 or (L_rms_>10.0 and I_rms_>4.0) |
